# Myricanol Inhibits Platelet Derived Growth Factor-BB-Induced Vascular Smooth Muscle Cells Proliferation and Migration *in vitro* and Intimal Hyperplasia *in vivo* by Targeting the Platelet-Derived Growth Factor Receptor-β and NF-κB Signaling

**DOI:** 10.3389/fphys.2021.790345

**Published:** 2022-02-03

**Authors:** Siyuan Fan, Cheng Wang, Kai Huang, Minglu Liang

**Affiliations:** ^1^Cardiovascular Center, Liyuan Hospital, Tongji Medical College, Huazhong University of Science and Technology, Wuhan, China; ^2^Clinic Center of Human Gene Research, Union Hospital, Tongji Medical College, Huazhong University of Science and Technology, Wuhan, China; ^3^Department of Cardiology, Union Hospital, Tongji Medical College, Huazhong University of Science and Technology, Wuhan, China

**Keywords:** myricanol, vascular smooth muscle cells, PDGF-BB, PDGFRβ, intimal hyperplasia

## Abstract

The abnormal proliferation and migration of Vascular smooth muscle cells (VSMCs) are related to many cardiovascular diseases, including atherosclerosis, restenosis after balloon angioplasty, hypertension, etc. Myricanol is a diarylheptanoid that can be separated from the bark of Myrica rubra. It has been reported that myricanol can anti-inflammatory, anti-cancer, anti-neurodegenerative, promote autophagic clearance of tau and prevent muscle atrophy. But its potential role in the cardiovascular field remains unknown. In this study, we investigated the effect of myricanol on the proliferation and migration of VSMCs *in vitro* and on the intimal hyperplasia *in vivo*. *In vitro* experiments, we found myricanol can inhibit the proliferation and migration of VSMCs induced by PDGF-BB. In terms of mechanism, the preincubation of myricanol can suppress the PDGF-BB induced phosphorylation of PDGFRβ and its downstream such as PLCγ1, Src, and MAPKs. In addition, NF-kB p65 translocation was also suppressed by myricanol. *In vivo* experiments, we found myricanol can suppress the intimal hyperplasia after wire ligation of the carotid artery in mice. These results may provide a new strategy for the prevention and treatment of coronary atherosclerosis and post-stent stenosis in the future.

## Introduction

VSMCs are an important part of the blood vessel wall. The abnormal proliferation and migration of VSMCs are related to many diseases, including atherosclerosis, restenosis after balloon angioplasty, hypertension, etc. (Owens et al., 2004; Bennett et al., 2016). Therefore, it is necessary to inhibit abnormal proliferation and migration of VSMCs for the treatment of many diseases.

A variety of growth factors, signal molecules, and transcription factors regulates the proliferation and migration of VSMCs, in which the PDGFRβ-mediated pathway plays an important role (Owens et al., 2004). Platelet-derived growth factor receptor-β (PDGFRβ) is a typical receptor tyrosine kinase, whose natural ligand is platelet Derived Growth Factor-BB (PDGF-BB) (Fredriksson et al., 2004). PDGF-BB is also one of the most powerful mitogens and chemokines of VSMC and occupies an important place in a variety of vascular diseases (Ferns et al., 1991; Ross, 1993; Pompili et al., 1995; Schwartz, 1997; Heldin and Westermark, 1999). Once PDGF-BB binds PDGFRβ, many pathways [such as Src, PLCγ1 and mitogen-activated protein kinases (MAPKs)] will be activated, which promotes the proliferation and migration of cells (Andrae et al., 2008).

Myrica rubra is a traditional crop mainly grow in China and Southeast Asia, whose bark is Chinese traditional medicine used for burns and skin diseases (Kim et al., 2013). Recently, researchers have reported that several chemical constituents isolated from the bark of Myrica rubra are well recognized for their medicinal values (Shen et al., 2019), such as quercetin, dihydromyricetin, and myricetin. Myricanol is a diarylheptanoid (Yoshimura et al., 2012) that can be separated from the bark of Myrica rubra by a systematic method (Inoue, 1993). According to recent researches, myricanol has a wide variety of bioactivities such as anti-inflammatory, anti-cancer, anti-neurodegenerative, promote autophagic clearance of tau and prevent muscle atrophy (Shen et al., 2019). But its potential role in the cardiovascular field remains unknown.

Therefore, in this study, we investigated the effect of myricanol on the proliferation and migration of VSMCs *in vitro* and *in vivo*. *In vitro*, we investigated the effect of myricanol on PDGF-BB-induced proliferation and migration of VSMCs. *In vivo*, we investigated the effect of myricanol on intimal hyperplasia induced by carotid artery ligation in mice.

## Materials and Methods

### Reagents

Myricanol was purchased from ChemFaces (Wuhan, China). Dulbecco’s Modified Eagle’s Medium (DMEM) and fetal bovine serum (FBS) were purchased from GIBCO. Recombinant human PDGF-BB was purchased from Corning Incorporated. EdU kit was purchased from Ribobio. Antibodies of the total levels and phosphorylation of PDGFRα, PDGFRβ, PLCγ1, Src, ERK1/2, JNK, p38, Rb, and p65 were purchased from Cell Signaling Technology. Anti-metalloproteinase 2 (MMP2), anti-matrix metalloproteinase 9 (MMP9), anti-Cyclin D1 (CCND1), and anti-Cyclin E1 (CCNE1) were purchased from Abcam. PCNA, E2F1, P21, P27, P53, Caspase 3, BAX, BCL2, and GAPDH were purchased from proteintech.

### Cell Culture

Primary VSMCs were isolated from the thoracic aortas of SD rats weighing 150–180 g in an enzymatical way. Rat aortic arteries were removed under sterile conditions. Adventitia from the aorta was removed under a dissecting microscope. The aorta was cut into pieces approximately 1–2 mm sections and digested with Enzyme solution (collagenase type II 3 mg/ml and elastase 1 mg/ml) for 2 h. Then cells were cultured in a flask with DMEM supplemented with 15% FBS, and passage 3–6 were used for experiments (Supplementary Figure 1).

### EdU Incorporation Assay

In starvation conditions, VSMCs were treated with indicated concentrations myricanol or not for 30 min, then VSMCs were treated with PDGF-BB (30 ng/ml) for 12 h and cultured into 96-well plates (3 × 10^3^ cells/well). After incubation with a medium containing EdU for another 2 h, the cells were fixed with 4% paraformaldehyde, and EdU Incorporation Assay was performed according to the manufacturer’s instructions. Images were photographed by using Olympus cellSens Entry.

### Migration Assay

For the wound healing assay, VSMCs in a 6-well plate with about 90% confluence were treated with indicated concentrations myricanol or not for 30 min in starvation conditions, then VSMCs were treated with PDGF-BB (30 ng/ml). Cell monolayers were scratched by a 200-μl pipette tip and photos were taken under a microscope (Olympus) at 0 and 24 h.

For transwell migration assay, VSMCs were treated with indicated concentrations myricanol or not for 30 min in starvation conditions, then VSMCs were treated with PDGF-BB (30 ng/ml). Then 1 × 10^5^ VSMCs were detached and suspended into a transwell upper surface with 200 μl of DMEM medium with FBS free. 500 μl of FBS-free DMEM medium with PDGF-BB (30 ng/ml) was added in the lower chamber. After 24 h incubation at 37°C, the migrated cells were fixed in 4% paraformaldehyde for 30 min, stained in 0.1% crystal violet for 15 min and photographed by microscope for six randomly assigned fields (Olympus).

### Western Blot

The VSMCs were cultured into six-well plates up to 80% confluence. After being pretreated with indicated concentrations of myricanol or vehicle for 30 min, the cells were stimulated by PDGF-BB (30 ng/ml) for 5, 15, 60 min or another 24 h. Western blotting was performed following procedures described previously (Wang et al., 2019).

### Nuclear and Cytoplasmic Extracts

Primary VSMCs were seeded in a round cell culture dish with a diameter of 60 mm. After the density reached 80% confluence, the cells were preadministrated with vehicle or myricanol at a concentration of 30 μM, and PDGF-BB was administered 30 min later. After 4 h, a nuclear protein and cytoplasmic protein extraction kit (Beyotime, P0027) was used to extract the nuclear and cytosolic proteins.

### Immunofluorescence Analysis

Primary VSMCs were seeded in a round cell culture dish with a diameter of 15 mm. The cells were preadministrated with vehicle or myricanol at a concentration of 30 μM, and PDGF-BB was administered 15 min later. The cells were fixed in 4% formaldehyde for 30 min and immunostained with p65 (CST, #8242) antibodies overnight at 4°C, then incubated with the indicated secondary antibodies for 1 h at 37°C. Nuclei were stained with DAPI for 20 min at 37°C. Photos were taken under a fluorescence microscope (Olympus).

### Carotid Artery Wire Ligation Injury Model

Animal housing and procedures were approved by the Institutional Animal Care and Use Committee (IACUC) of Huazhong University of Science and Technology, and IACUC number is 2561. Surgery was performed under sterile conditions. Eight-week-old male C57BL/6 mouses were anesthetized with an intraperitoneal injection of pentobarbital sodium. In ligated animals (*n* = 6), the left common carotid artery was dissected from the surrounding tissue under a microscope and ligated by using 6–0 silk ligature. The right common carotid artery was dissected without ligation as a sham surgery. Then, mice were randomly divided into two groups on average. Myricanol (5 mg/kg/day) or hydration medium (PEG 400) was intraperitoneally injected into C57BL/6 mice for 14 days. Then mice were euthanized and common carotid arteries were excised. After being fixed with 4% formaldehyde and embedded in paraffin, Cross-sections of the common carotid artery were stained with hematoxylin and eosin (H&E) and elastic Masson trichrome solutions.

### Statistical Analysis

SPSS v.20 was used for statistical analyses. All experiments were performed at least three times, and data are given as means ± SEM. The two-tailed unpaired Student’s *t*-test was used for comparisons of two groups and differences were considered significant at *P* < 0.05.

## Results

### Myricanol Inhibits the Proliferation of Vascular Smooth Muscle Cells Induced by Platelet Derived Growth Factor-BB

**FIGURE 1:**
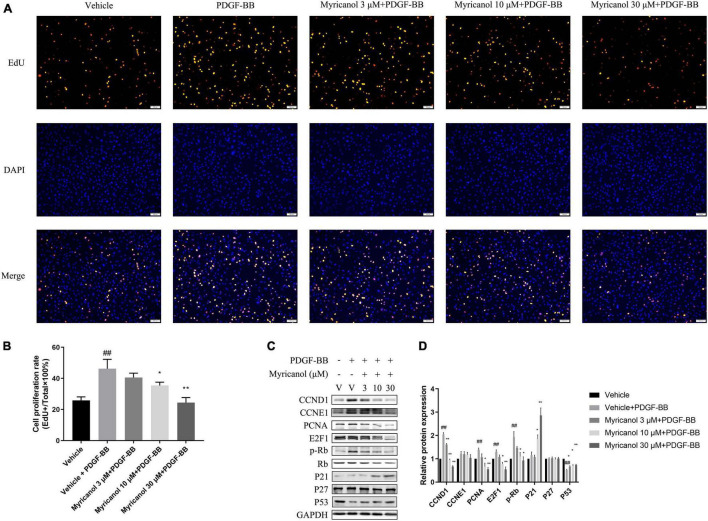
Myricanol inhibits PDGF-BB induced VSMCs proliferation. **(A)** VSMCs were stained with EdU (red) and DAPI (blue). Scale bar, 100 μm. **(B)** Quantification of the proliferative cells (normalized to vehicle). Data are represented as mean ± SEM (*n* = 3). ^##^*P* < 0.01 vs. the Vehicle group. *P < 0.05, **P < 0.01 vs. the Vehicle + PDGF-BB group. **(C)** After being pretreated with indicated concentrations of myricanol or vehicle for 30 min, the cells were stimulated by PDGF-BB (30 ng/ml) for 24 h. The protein level of CCND1, CCNE1, PCNA, E2F1, P21, P27, P53, Rb, and Phosphorylated Rb were determined by Western blot analysis. **(D)** Quantification of the Western blot analysis. Data are represented as mean ± SEM (*n* = 3). ^##^*P* < 0.01 vs. the Vehicle group. *P < 0.05, **P < 0.01 vs. the Vehicle + PDGF-BB group.

When VSMCs are activated by various injury stimuli, they change from a resting state to a proliferative phenotype and migrate under the intima. To investigate the effect of myricanol on PDGF-BB-induced VSMCs proliferation, we performed a EdU assay. The results showed that the ability of cell proliferation was significantly improved under the stimulation of PDGF-BB and was apparently inhibited by myricanol. We found that the effect of myricanol on VSMC proliferation is dose-dependent. For example, 3 μM of myricanol treatment showed no significant influence, whereas both 10 and 30 μM of myricanol can significantly suppressed the proliferation of VSMCs (Figures 1A,B).

Under the action of extracellular stimulus, the expression of contractile protein in VSMC is significantly reduced, while the expression of cell cycle-related proteins increases rapidly. As shown in Figures 1C,D, the protein level of PCNA, E2F1, phosphorylated Rb, Rb, CCND1, CCNE1, p21, p27, and p53 were detected by western blot analysis. We find that CCND1, CCNE1, PCNA, E2F1, and p-Rb were significantly increased by PDGF-BB stimulation, whereas the myricanol treatment can partially or completely block the effects. Meanwhile, p53 was significantly inhibited by PDGF-BB stimulation, whereas the myricanol treatment can partially recover the effects. Different from the above results, PDGF-BB stimulation had no effect on the expression of p21 and p27, while preadministrated of myricanol increased the expression of p21.

To further confirm the effect of myricanol on cell apoptosis and necrosis, western blot and LDH assays were performed. Western blot results showed that PDGF-BB and myricanol had no effect on the protein level of caspase 3, cleaved caspase3, BAX and BCL2. LDH assay showed that PDGF-BB and myricanol had no effect on LDH release (Supplementary Figure 2). These results indicated that myricanol have no effect on cell apoptosis and necrosis.

### Myricanol Inhibits the Migration of Vascular Smooth Muscle Cells Induced by Platelet Derived Growth Factor-BB

**FIGURE 2:**
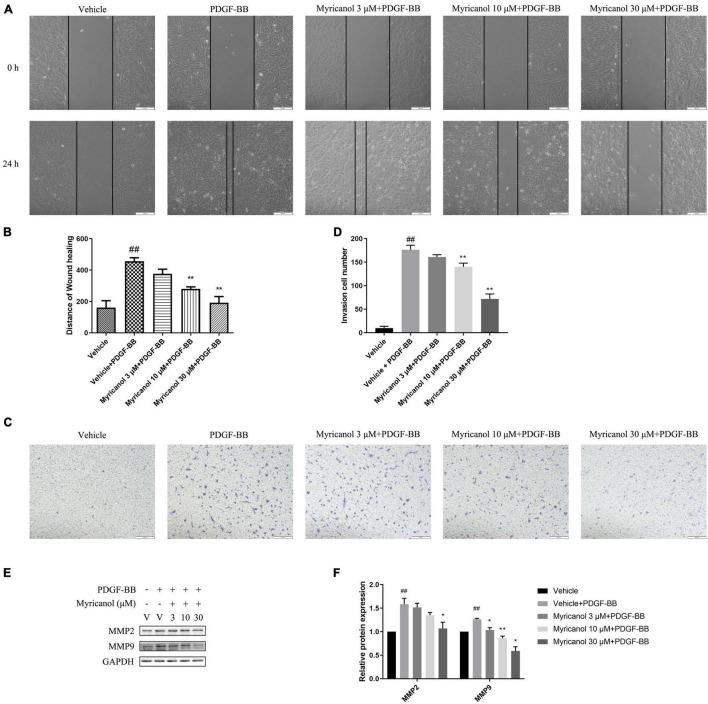
Myricanol inhibits VSMCs migration. **(A)** In starvation conditions, cells were treated with indicated concentrations myricanol or not for 30 min, then scratched and treated with PDGF-BB (30 ng/ml) for 24 h. Scale bar, 100 μm. **(B)** Quantification of the area of wound closure (%). Data are represented as mean ± SEM (*n* = 3). ^##^*P* < 0.01 vs. the Vehicle group. **P < 0.01 vs. the Vehicle + PDGF-BB group. **(C)** After treated with PDGF-BB and myricanol for 24 h, a transwell migration assay of VSMCs was performed and representative images were shown. Scale bar, 100 μm. **(D)** Quantification of migrated cells. Data are represented as mean ± SEM (*n* = 3). ^##^*P* < 0.01 vs. the Vehicle group. **P < 0.01 vs. the Vehicle + PDGF-BB group. **(E)** After being pretreated with indicated concentrations of myricanol or vehicle for 30 min, the cells were stimulated by PDGF-BB (30 ng/ml) for 24 h. The protein level of MMP2 and MMP9 were determined by Western blot analysis. **(F)** Quantification of the Western blot analysis. Data are represented as mean ± SEM (*n* = 3). ^##^*P* < 0.01 vs. the Vehicle group. *P < 0.05, **P < 0.01 vs. the Vehicle + PDGF-BB group.

To investigate whether myricanol was able to affect the migration in VSMCs, We then assessed the effect by wound healing assay and transwell migration assay (Figures 2A,C). The wound healing assay result showed that 10 and 30 μM myricanol but not 3 μM of myricanol could partially suppress the migration induced by PDGF-BB stimulation, and the result of the transwell migration assay seems to be similar. The migration of VSMCs was shown by the distance of the gap in the wound healing assay (Figure 2B) and the migration cell number in the transwell migration assay (Figure 2D).

In the process of intimal hyperplasia, a large number of proteoglycans and extracellular matrix remodeling related proteins (such as MMP2, MMP9) are synthesized and secreted to promote the migration of VSMCs. Therefore, the protein levels of migration-associated protein MMP2 and MMP9 were detected by western blot analysis, which increased by PDGF-BB stimulation. However, the effect on MMP2 and MMP9 seems to be different. The increasing of MMP9 protein level was significantly suppressed at 3, 10, and 30 μM myricanol, however, increasing of MMP2 protein level can only be significantly suppressed at 30 μM myricanol (Figures 2E,F). Further, consistent with the above results, the activity of MMP2 and MMP9 were increased by PDGF-BB stimulation, while the myricanol treatment can partially or completely block the effects as shown in Zymography assays (Supplementary Figure 3).

### Myricanol Inhibits the Activation of Platelet-Derived Growth Factor Receptor Pathway and NF-κB p65 Translocation Induced by Platelet Derived Growth Factor-BB

**FIGURE 3:**
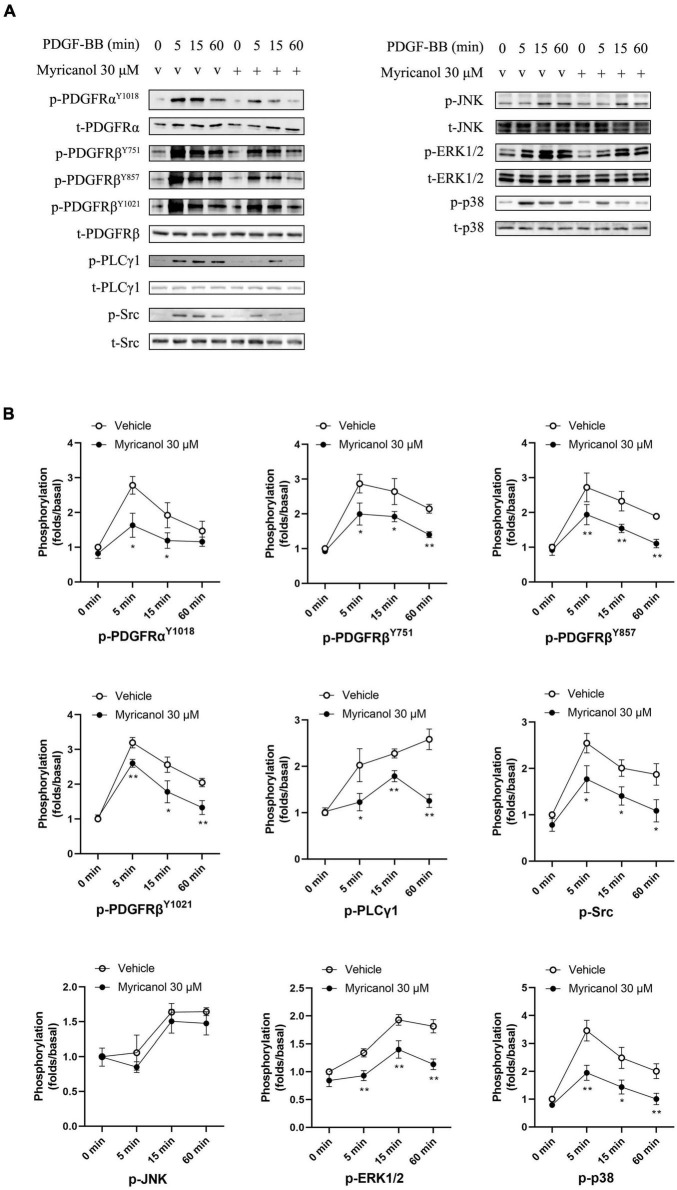
Effect of myricanol on the phosphorylation of PDGFRβ, PLCγ1, Src and downstream MAPKs. **(A)** The protein level of phosphorylated PDGFRα, PDGFRβ, PLCγ1, Src, JNK, ERK1/2 and p38 were determined by Western blot analysis. **(B)** Quantification of the Western blot analysis. Data are represented as mean ± SEM (*n* = 3). *P < 0.05, **P < 0.01 vs. the Vehicle + PDGF-BB group.

At present, it is known that a variety of cell pathways and their effector molecules in VSMC have undergone significant changes, and they jointly participate in the coordinated regulation of VSMC proliferation and migration, of which PDGFRβ pathway is the most important (Levitzki, 2005; Wang Y. et al., 2020). To investigate how myricanol influences the proliferation and migration of VSMC induced by PDGF-BB, we tested if myricanol could suppress the phosphorylation of PDGFRα, PDGFRβ and its downstream PLCγ1, Src and MAPK in VSMC. VSMCs were treated with vehicle or 30 μM myricanol 30 min before exposure to PDGF-BB, and then cells were stimulated by PDGF-BB and the cytolytic products were harvested at different time point after stimulation (5 min, 15 min, 60 min). The expression of p-PDGFRα^Y1018^, p-PDGFRβ^Y751^, p-PDGFRβ^Y857^, p-PDGFRβ^Y1021^, p-PLCγ1, p-Src, p-JNK, p-ERK1/2, and p-p38 were increased after PDGF-BB stimulation in 5, 15, and 60 min, and pretreatment with 30 μM myricanol repressed the phosphorylation of these proteins (except p-JNK) at the same time points after PDGF-BB stimulation (Figure 3). The specific inhibitors for PDGFRβ, JNK, ERK1/2 and p38 were provided to verify the effect of myricanol. The results showed that myricanol has weaker inhibitory effect on PDGFRα^Y1018^, p-PDGFRβ^Y751^, p-PDGFRβ^Y857^, p-PDGFRβ^Y1021^, p-ERK1/2 and p-p38 than PDGFRβ, JNK, ERK1/2 and p38 inhibitors (Supplementary Figure 4).

**FIGURE 4:**
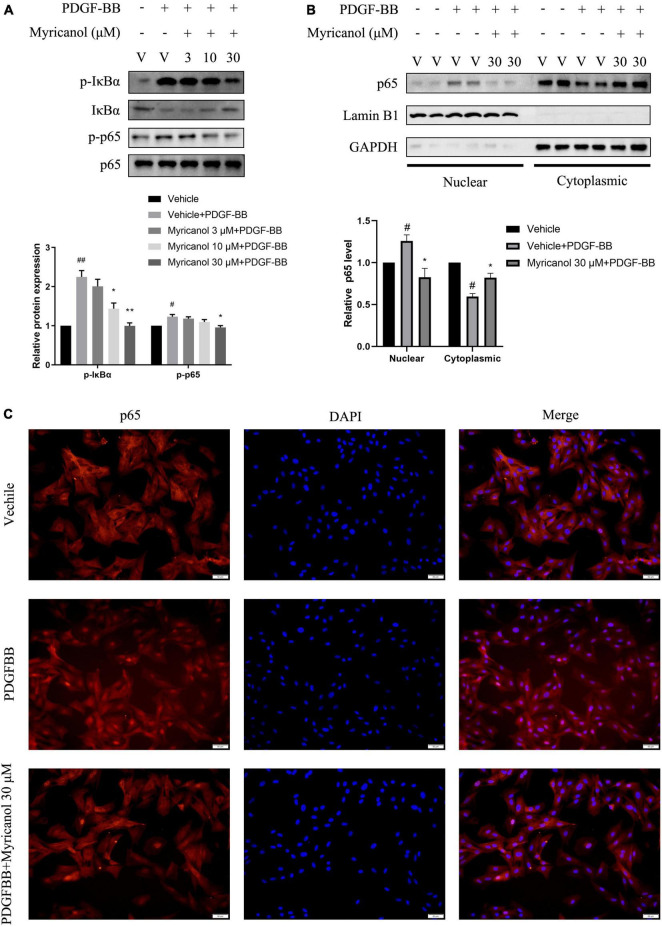
Effects of myricanol on PDGF-BB-induced NF-κB signaling in VSMCs. **(A)** After being pretreated with indicated concentrations of myricanol or vehicle for 30 min, the cells were stimulated by PDGF-BB (30 ng/ml) for 5 min. The protein level of p-IκBα, IκBα were determined by Western blot analysis. After being pretreated with indicated concentrations of myricanol or vehicle for 30 min, the cells were stimulated by PDGF-BB (30 ng/ml) for 30 min. The protein level of p-p65 and p65 were determined by Western blot analysis. **(B)** p65 nuclear translocation was analyzed by Western blot analysis. **(C)** p65 nuclear translocation was analyzed by immunocytochemistry. Scale bar, 50 μm. Data are represented as mean ± SEM (*n* = 3). ^#^*P* < 0.05, ^##^*p* < 0.01 vs. the Vehicle group. *P < 0.05, **p < 0.01 vs. the Vehicle + PDGF-BB group.

In addition, NF-κB is a critical signaling in VSMC proliferation and migration (Lu et al., 2018). Our results exhibited that myricanol can inhibit the phosphorylation level of IκBα and p65 and the nuclear translocation of p65 stimulated by PDGF-BB in VSMC (Figure 4).

### Myricanol Inhibits the Neointimal Hyperplasia Induced by Carotid Artery Ligation

**FIGURE 5:**
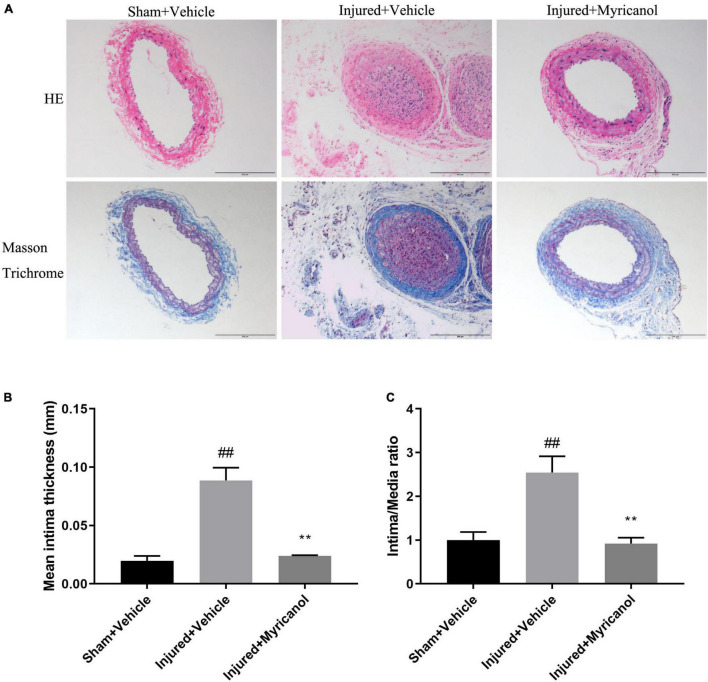
Effect of myricanol on carotid artery ligation induced neointimal hyperplasia for 14 days. **(A)** myricanol (5 mg/kg/day) or hydration medium (PEG 400) was intraperitoneally injected for 14 days after carotid artery ligation. H&E-stained and elastic Masson trichrome-stained sections of all groups were shown. Scale bar, 200 μm. **(B)** Quantification of the mean intimal thickness. Data are represented as mean ± SEM (*n* = 3). ^##^*P* < 0.01 vs. the Sham + Vehicle group. **P < 0.01 vs. the Injured + Vehicle group. **(C)** Quantification of the ratio of intima to media (I/M ratio). Data are represented as mean ± SEM (*n* = 3). ^##^*P* < 0.01 vs. the Sham + Vehicle group. **P < 0.01 vs. the Injured + Vehicle group.

To assess the effect of myricanol on intimal hyperplasia after vascular injury, we used the carotid artery wire ligation model. Myricanol (5 mg/kg/day) or hydration medium (PEG 400) was intraperitoneally injected for 14 days after carotid artery ligation. Comparing with the Sham surgery group, the ligation surgery group showed that intimal hyperplasia was well developed. The sections stained with elastic Masson trichrome solutions were used to highlight the media (Figure 5A). Comparing with the ligation surgery group, the myricanol treated ligation surgery group showed significantly reduced intima, both the ratio of intima to media (I/M ratio) and intimal area (Figures 5B,C). Immunofluorescence staining showed that the myricanol-treated ligation surgery group had significantly inhibited Ki67 expression (Supplementary Figures 5A,B). In addition, macrophages are known to play a role in the progression of intimal hyperplasia and other cardiovascular disorders (Zhang et al., 2021). Our results exhibited that the expression of F4/80 significantly increased in the ligation surgery group comparing with the sham surgery group, While the myricanol treatement significantly decreased F4/80 expression (Supplementary Figures 5C,D). These results indicate that myricanol significantly diminishes the neointimal hyperplasia induced by carotid artery ligation.

## Discussion

Intimal hyperplasia is a common physiological feature of cardiovascular diseases such as atherosclerosis and restenosis after angioplasty, and it is closely related to vascular remodeling. Preventing intimal hyperplasia and postoperative restenosis has always been an important research topic worldwide. Our study for the first time investigated the effect of myricanol on the proliferation and migration of VSMCs *in vitro* and on the intimal hyperplasia *in vivo*, which suggested the therapeutic potential of myricanol in cardiovascular disease.

PDGF-BB had been proved that it can significantly improve the proliferation and migration of VSMCs *in vitro*, playing an important role in the development of many cardiovascular diseases such as atherosclerosis and restenosis (Zhan et al., 2003). Therefore, we studied whether it will be affected by myricanol that the proliferation and migration of VSMCs induced by PDGF-BB.

Firstly the EDU assay showed that myricanol could inhibit the proliferation of VSMCs, and wound healing assay and transwell migration assay showed that myricanol could inhibit the migration of VSMCs. Then, Western blot assay was used to measure the protein levels of cell proliferation and migration marker genes. Compared to the vehicle + PDGF-BB group, the PDGF-BB + Myricanol (10 and 30 μM) treated group showed significantly lower protein levels of PCNA, E2F1, Phosphorylated Rb, and MMP9. In the early G1 phases cell cycle, the activated cyclin-dependent kinase complexes (CDKCs) are formed by the binding of CCND and CDK4/6. Then CDKCs phosphorylate Rb protein to release E2F, which leads to the transcription of E2F target genes and promotes the G1/S transition (Duronio and Xiong, 2013). We found that myricanol can reduce the protein level of CCND1 and the downstream Rb Phosphorylation, which may lead to the repression of VSMC proliferation. It is reported that the cell proliferation induced by the MAPK pathway is closely bound up with CCND1. MAPKs such as ERK1/2 and JNK can activate c-JUN, which binds to the CCND1 promoter and functions in activating transcription of CCND1 (Wee and Wang, 2017). Inhibition of ERK1/2 by curcumin and PD98059, and JNKs by SP600125 could reduce the induction of CCND1 (Qin et al., 2014; Wang H. et al., 2020). MAPKs such as ERK1/2 and p38 also play important roles in the regulation of MMP9 expression. Completely inhibiting of either p38 or ERK1/2 alone or both of them can totally downregulate MMP9 expression (Cho et al., 2000). All these results suggest that the MAPK pathway may be related to the repression of proliferation and migration induced by myricanol. Therefore, we investigated the effect of myricanol on the MAPK pathway and the upstream PDGFRβ pathway.

The signal of PDGF-BB is transmitted by PDGFRβ. Once PDGFRβ is bound to PDGF-BB, it will experience autophosphorylation and activate downstream signal pathways such as Src, PLC, and MAPK, which promote PDGF-BB induced proliferation and migration of VSMC (Zhan et al., 2003; Andrae et al., 2008). Mitogen-activated protein kinases (MAPKs) play an important role in the proliferation and migration of VSMCs induced by PDGF-BB (Andrae et al., 2008), which is known as one of the most famous pathways regulating many cell functions such as proliferation, gene expression, differentiation, and mitosis (Johnson and Lapadat, 2002). Our results showed that myricanol could suppress the proliferation and migration of VSMC by inhibiting the phosphorylation of PDGFRβ and its downstream PLCγ1, Src, and MAPKs including ERK1/2 and p38. These results are consistent with our previous speculation. In addition, we also tested the effect of myricanol on NF-κB signal pathway stimulated by PDGF-BB, and the results showed that myricanol can also inhibit the phosphorylated level of p65 and the nuclear translocation of p65.

Many tyrosine kinase inhibitors can suppress the intimal hyperplasia after arterial injury by inhibiting the phosphorylation of PDGFRβ (Fishbein et al., 2000; Yu et al., 2001; Cheema et al., 2006; Masuda et al., 2011; Ishii et al., 2013). And p38, as a class of MAPKs, also plays an important role in vascular remodeling caused by balloon injury in rats (Zhan et al., 2003). These clues suggest that myricanol may have the potential to treat vascular proliferative diseases such as restenosis. Therefore, we assessed the effect of myricanol on intimal hyperplasia after carotid artery wire ligation and found that 14-day’ treatment of myricanol can significantly diminish the intimal hyperplasia. In addition, inflammatory cytokines produced by activated macrophages are the direct promoters of neointimal formation (Zhang et al., 2021). And our results showed that myricanol can also inhibit the macrophage infiltration after carotid artery wire ligation. So myricanol inhibits the proliferation and migration of smooth muscle cells and the infiltration of macrophages, thereby reducing intimal hyperplasia.

## Conclusion

In summary, our study illustrates the effect of myricanol on the proliferation and migration of VSMCs both *in vitro* and *in vivo* experiments. On the one hand, myricanol can inhibit the proliferation and migration of VSMC by suppressing many signaling pathways, including PDGFRβ and NF-κB signaling. On the other hand, Myricanol can suppress the intimal hyperplasia after wire ligation of the carotid artery in mice. These results may provide a new strategy for cardiovascular diseases caused by the abnormal proliferation of VSMC.

## Data Availability Statement

The raw data supporting the conclusions of this article will be made available by the authors, without undue reservation.

## Ethics Statement

The animal study was reviewed and approved by the Local Animal Care and Use Committee of Huazhong University of Science and Technology.

## Author Contributions

ML and KH conceived, designed the experiments, prepared, and revised the manuscript. SF and CW performed the experiments and prepared the manuscript. All authors gave final approval.

## Conflict of Interest

The authors declare that the research was conducted in the absence of any commercial or financial relationships that could be construed as a potential conflict of interest.

## Publisher’s Note

All claims expressed in this article are solely those of the authors and do not necessarily represent those of their affiliated organizations, or those of the publisher, the editors and the reviewers. Any product that may be evaluated in this article, or claim that may be made by its manufacturer, is not guaranteed or endorsed by the publisher.
