# Supplementary material for: Myricanol Inhibits Platelet Derived Growth Factor-BB-Induced Vascular Smooth Muscle Cells Proliferation and Migration in vitro and Intimal Hyperplasia in vivo by Targeting the Platelet-Derived Growth Factor Receptor-β and NF-κB Signaling
Source: Front Physiol. 2022 Feb 3;12:790345. doi: 10.3389/fphys.2021.790345 (PMC8850918; doi:10.3389/fphys.2021.790345)
Supplement: Supplementary file 1 [file Data_Sheet_1.PDF]

Materials and methods

Immunofluorescence analysis

For cells cultured in plates, cells were fixed in 4% formaldehyde for 30 minutes and immunostained with α-SMA (proteintech 14395-1-AP) antibodies overnight at 4°C, then incubated with the indicated secondary antibodies for 1 hours at 37°C. Nuclei were stained with DAPI for 20 minutes at 37°C. Photos were taken under a fluorescence microscope (Olympus).

For sections harvested from animal model, the paraffin-embedded common carotid arteries were sectioned in increments of 6 μm and immunostained with Ki67 (ab15580; Abcam) and F4/80 antibodies (ab5694; Abcam) overnight at 4°C , then incubated with the indicated secondary antibodies for 1 hours at 37°C. Nuclei were stained with DAPI for 20 minutes at 37°C. Photos were taken under a fluorescence microscope (Olympus).

LDH assay

The level of LDH in cell culture supernatants was assayed by using LDH Cytotoxicity Assay Kit（beyotime C0016) according to the manufacturer's instructions. In brief, VSMCs were cultured in 96-well culture plates (2×10^4 cells/well) and treated with indicated concentrations of Myricanol or vehicle for 24 h. Then 60 μl cell-free supernatant was incubated with 120 μl LDH substrate solution for 30 min, and the absorbance at 490 nm was measured by using a microplate spectrophotometer. The LDH release rate was calculated: [ODsample－ODblank]/[ODTriton X-100－ODblank].

Zymography assay

The VSMCs were cultured into six-well plates at a density of 1×10^6 cells/well. After being pretreated with indicated concentrations of Myricanol or vehicle for 30 min in starvation conditions, the cells were stimulated by PDGF-BB (30 ng/ml) for 24 h. Culture supernatants were collected and centrifuged at 9700 g for 1 min at 4 °C. Same amount of supernatants (25 μL) of each group were prepared for next step. Then

Zymography assay were performed following the manufacturer's instructions of the MMP Zymography assay kit (Applygen P1700).

Figures

Supplementary Figure 1 Immunofluorescence staining of α-SMA in Primary VSMCs. Primary VSMCs were isolated from the thoracic aortas of SD rats. VSMCs were stained with α-SMA (red) and DAPI (blue). Scale bar, 20 μm

Supplementary Figure 2 Effects of Myricanol on PDGF-BB-induced apoptosis and necrosis assays in VSMCs. (A) After being pretreated with indicated concentrations of myricanol or vehicle for 30 min, the cells were stimulated by PDGF-BB (30 ng/ml) for 24 h. The protein level of Caspase 3, Cleaved caspase 3, BAX and BCL2 were determined by Western blot analysis. Data are represented as mean±SEM (n=3) (B) VSMCs were treated with indicated concentrations of myricanol or vehicle for 24 h and LDH in cell culture supernatants was assayed. Data are represented as mean±SEM (n=4).

Supplementary Figure 3 Effects of Myricanol on PDGF-BB-induced MMP2 and MMP9 activity in VSMCs. The activity of MMP2 and MMP9 were detected by Zymography assays.

Supplementary Figure 4 The specific inhibitors for PDGFRα, PDGFRβ, JNK, ERK1/2 and p38 were provided to reveal the specificity of myricanol on PDGFRβ signaling pathways. (A) The protein level of the phosphorylation of PDGFRα， PDGFRβ and downstream MAPKs were determined by Western blot analysis， while the specific inhibitors for PDGFRα (AP24534) and PDGFRβ (SU11248) were provided. (B) The protein level of the phosphorylation of PDGFRβ and downstream MAPKs were determined by Western blot analysis, while the specific inhibitors for JNK (JNK-IN-8), ERK1/2 (PD98059) and p38 (SB203580) were provided. Data are represented as mean±SEM (n=3). *$P<0.05$, **$P<0.01$ versus

the Vehicle group.

Supplementary Figure 5 Effect of Myricanol on carotid artery ligation induced proliferation and macrophage infiltration. (A-B) Effect of Myricanol on proliferation in vivo was assayed by Immunofluorescence staining of Ki67 (red) and DAPI (blue). (C-D) Immunofluorescence staining for F4/80 (red) and DAPI (blue) was performed to analysis macrophage infiltration in vivo. Data are represented as mean±SEM (n=3). Scale bar, 50 μm. ##$P<0.01$ versus the Sham+Vehicle group. *$P<0.05$, **$P<0.01$ versus the Injured+Vehicle group.
