# Supplementary figures and images for: Myricanol Inhibits Platelet Derived Growth Factor-BB-Induced Vascular Smooth Muscle Cells Proliferation and Migration in vitro and Intimal Hyperplasia in vivo by Targeting the Platelet-Derived Growth Factor Receptor-β and NF-κB Signaling
Source: Front Physiol. 2022 Feb 3;12:790345. doi: 10.3389/fphys.2021.790345 (PMC8850918; doi:10.3389/fphys.2021.790345)

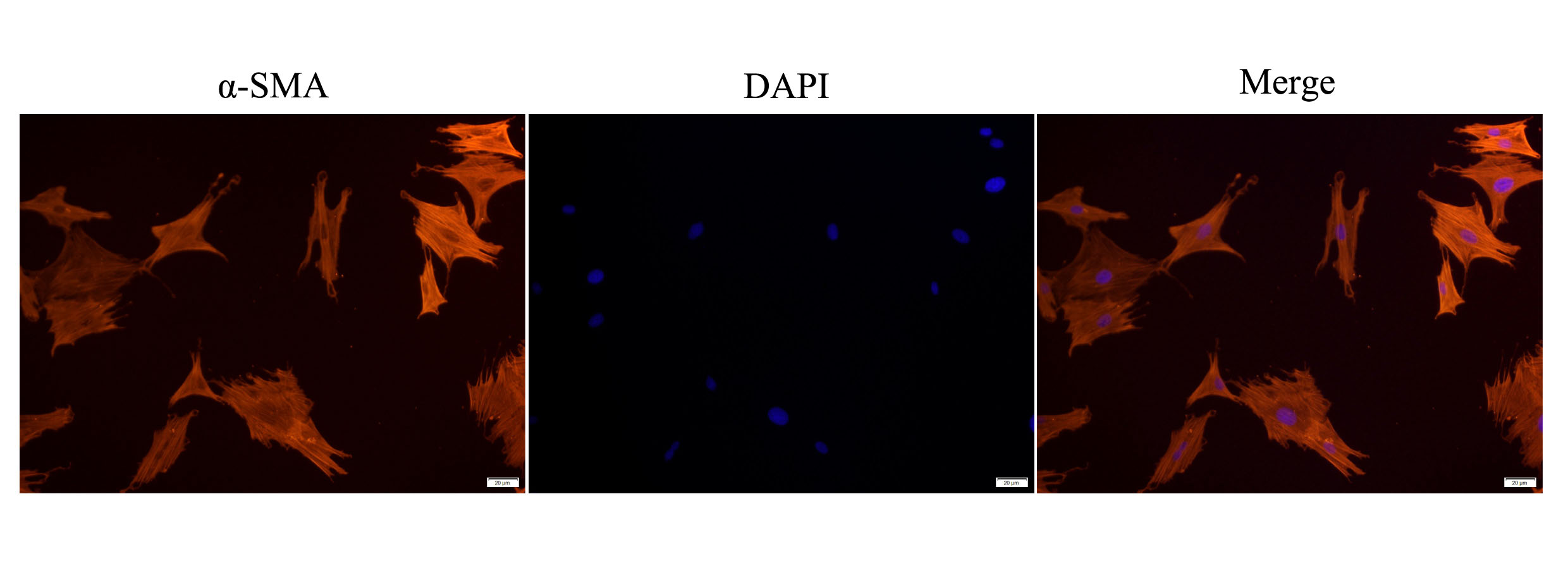

Supplement: Supplementary file 2 [file Image_1.JPEG]

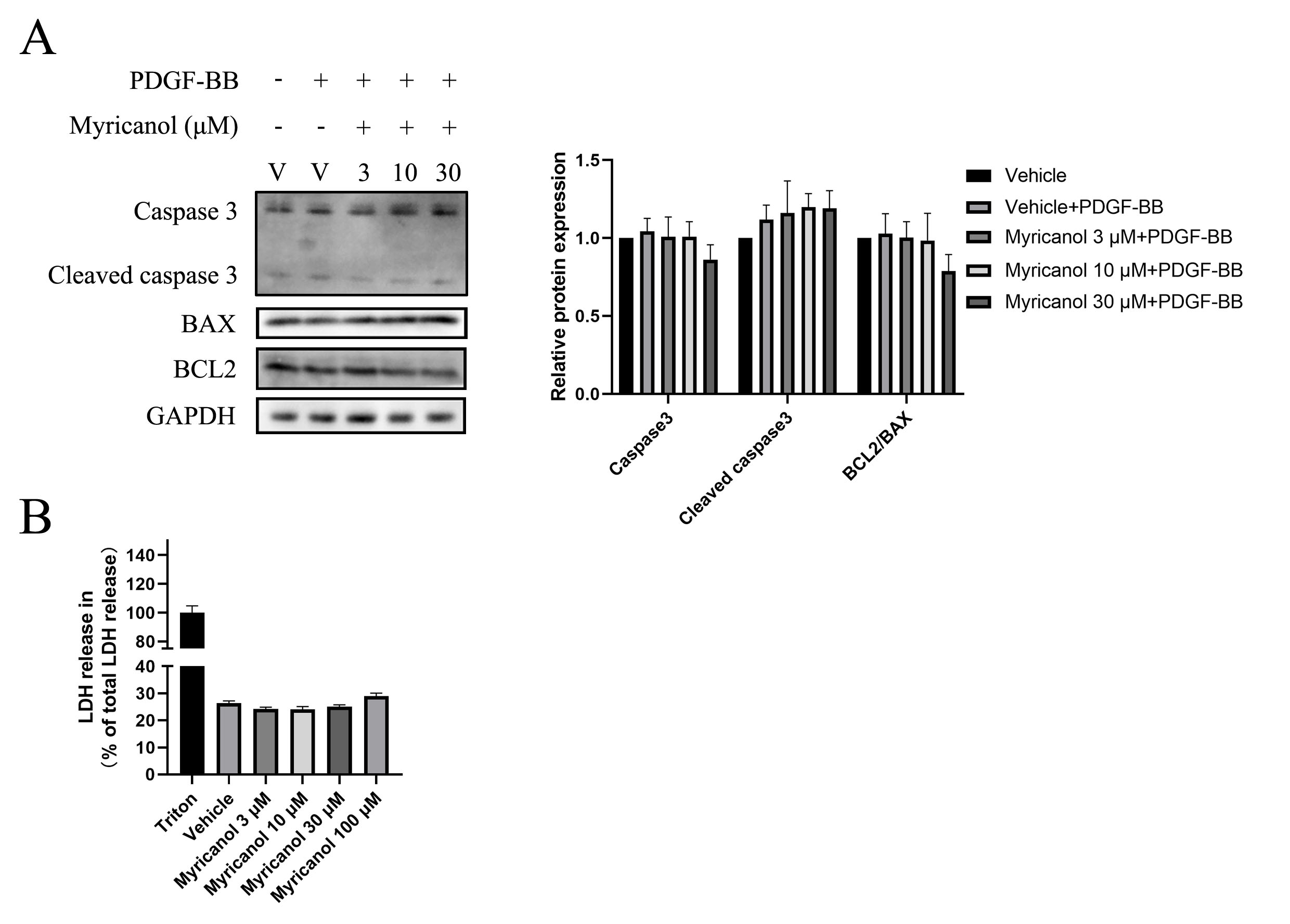

Supplement: Supplementary file 3 [file Image_2.JPEG]

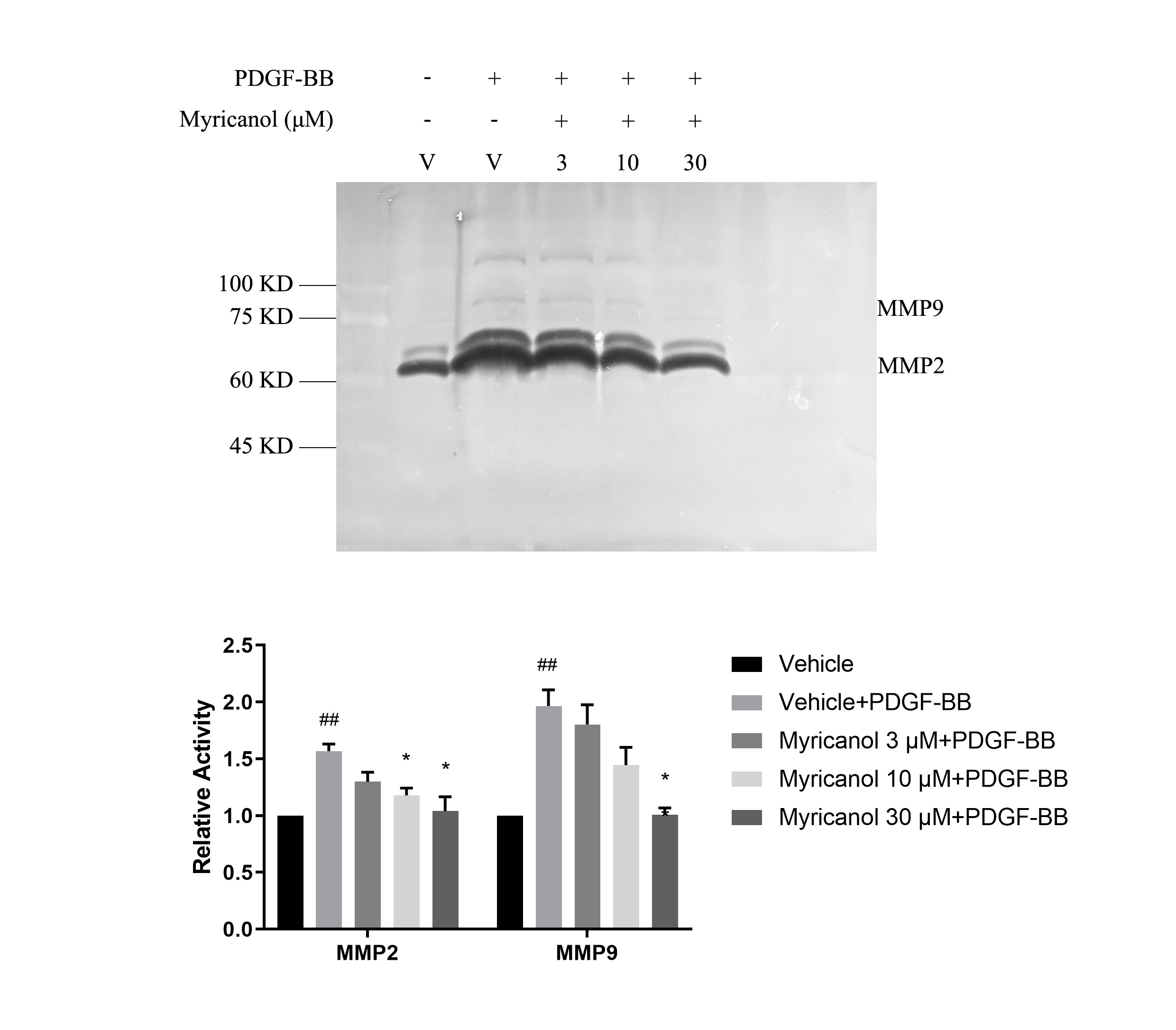

Supplement: Supplementary file 4 [file Image_3.JPEG]

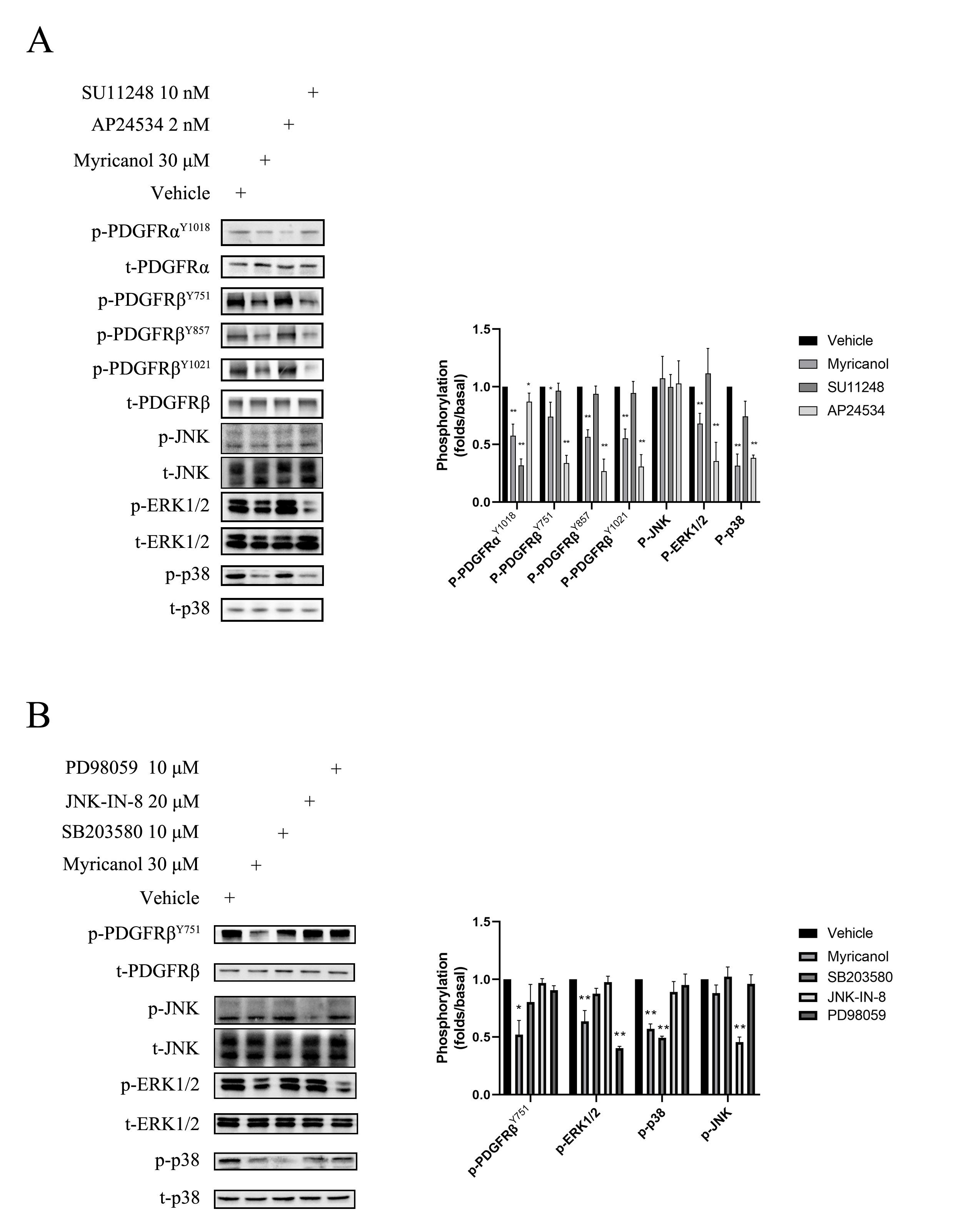

Supplement: Supplementary file 5 [file Image_4.JPEG]

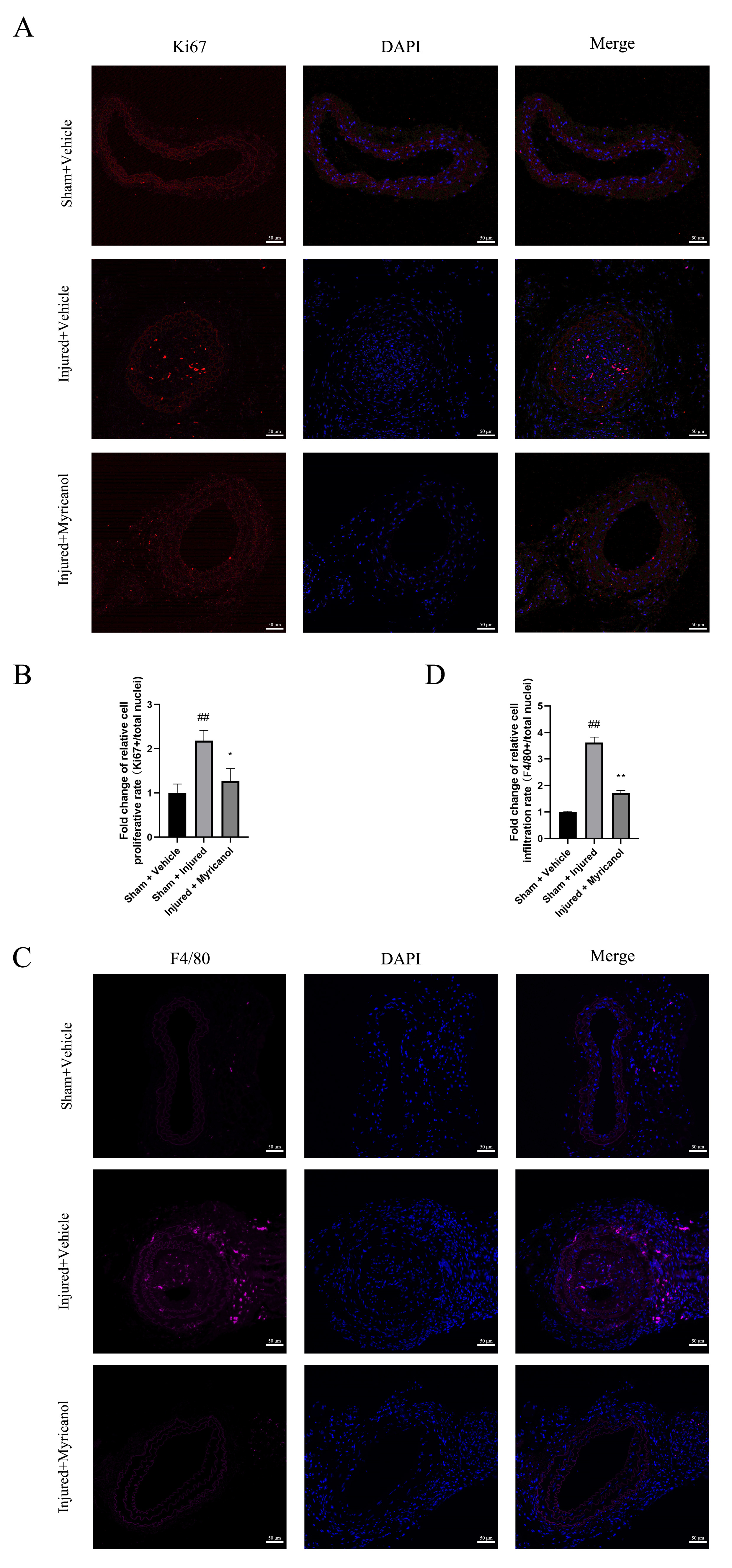

Supplement: Supplementary file 6 [file Image_5.JPEG]
